# The Structure, Activity, and Function of the SETD3 Protein Histidine Methyltransferase

**DOI:** 10.3390/life11101040

**Published:** 2021-10-02

**Authors:** Apolonia Witecka, Sebastian Kwiatkowski, Takao Ishikawa, Jakub Drozak

**Affiliations:** 1 Department of Metabolic Regulation, Institute of Biochemistry, Faculty of Biology, University of Warsaw, Miecznikowa 1, 02-096 Warsaw, Poland; aa.witecka@uw.edu.pl (A.W.); sp.kwiatkowski@uw.edu.pl (S.K.); 2 Department of Molecular Biology, Institute of Biochemistry, Faculty of Biology, University of Warsaw, Miecznikowa 1, 02-096 Warsaw, Poland

**Keywords:** SETD3, posttranslational modifications, protein histidine methylation, actin, polymerization, cytoskeleton, enteroviruses, oncogenesis

## Abstract

SETD3 has been recently identified as a long sought, actin specific histidine methyltransferase that catalyzes the *Nτ*-methylation reaction of histidine 73 (H73) residue in human actin or its equivalent in other metazoans. Its homologs are widespread among multicellular eukaryotes and expressed in most mammalian tissues. SETD3 consists of a catalytic SET domain responsible for transferring the methyl group from *S*-adenosyl-L-methionine (AdoMet) to a protein substrate and a RuBisCO LSMT domain that recognizes and binds the methyl-accepting protein(s). The enzyme was initially identified as a methyltransferase that catalyzes the modification of histone H3 at K4 and K36 residues, but later studies revealed that the only bona fide substrate of SETD3 is H73, in the actin protein. The methylation of actin at H73 contributes to maintaining cytoskeleton integrity, which remains the only well characterized biological effect of SETD3. However, the discovery of numerous novel methyltransferase interactors suggests that SETD3 may regulate various biological processes, including cell cycle and apoptosis, carcinogenesis, response to hypoxic conditions, and enterovirus pathogenesis. This review summarizes the current advances in research on the SETD3 protein, its biological importance, and role in various diseases.

## 1. Introduction

**Figure 1:**
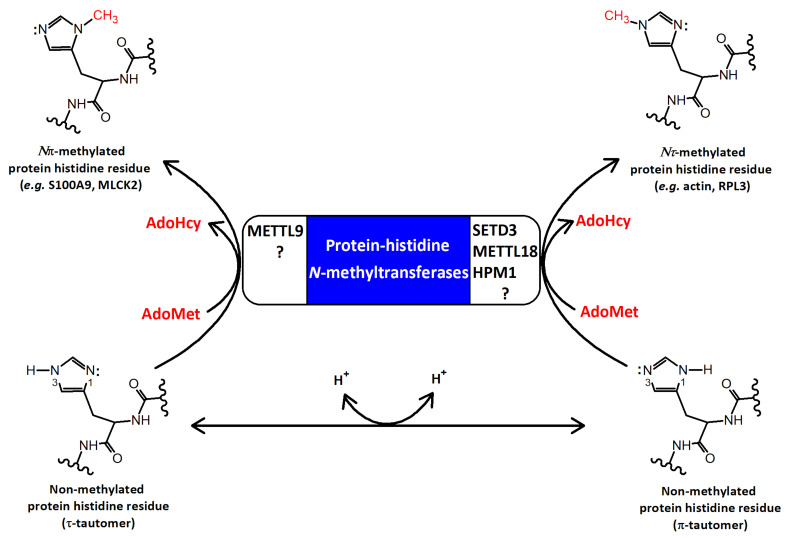
Reactions catalyzed by protein histidine *N*-methyltransferases. At pH ≈ 7, two neutral tautomers of histidine residues may exist in proteins: the *N*1-protonated π-tautomer and the *N*3-protonated τ-tautomer. Data show that different protein histidine methyltransferases catalyze the transfer of a methyl group from *S*-adenosyl-L-methionine (AdoMet) to specific nitrogen of the imidazole ring. HPM1, SETD3, METTL9, and METTL18 are the only enzymes characterized with this activity so far. AdoHcy—*S*-adenosyl-L-homocysteine.

One of the most common posttranslational modifications that modulates the physicochemical properties of proteins and determines their functional diversity, is the transfer of a methyl group from *S*-adenosyl-L-methionine (AdoMet) to their specific amino acid residues [1]. The primary target sites of methylation are lysine and arginine. However, this process may also occur on other amino acids, namely, cysteine, glutamate, glutamine, and histidine [2]. Decades of research into lysine and arginine methylation on histone tails have led to a fairly good understanding of the importance of such modifications in the epigenetic regulation of gene expression. Furthermore, it has become clear over time that a large number of nonhistone proteins may also be methylated at lysine and arginine residues, which may affect cellular physiology in mammals [2]. On the other hand, our knowledge about the mechanisms and biological significance of methylation on “noncanonical” amino acids has remained surprisingly limited. This seems particularly true for protein histidine. Histidine methylation on the *Nπ* or *Nτ* atom of the imidazole ring has been known for many years, but the process has so far been studied in greater detail only for a few proteins, including actin [3], S100A9 [4], myosin [5], MLCK2 [6], and RPL3 [7] (Figure 1). This fact is also indicated by the slow progress of research on actin histidine methylation.

The actin cytoskeleton, which is involved in a variety of central cellular processes, such as cell growth, division, and motility, has long been known to undergo different posttranslational modifications [8]. In 1967, Johnson and colleagues isolated actin from various vertebrate species, and demonstrated that *Nτ*-methylhistidine is a natural component of this protein and a product resulting from enzymatic methylation [9]. A similar finding was reported by Asatoor and Armstrong [10]. Later, attempts were made to determine the amino acid sequence around methylhistidine in skeletal muscle actin [11] and establish the biochemical importance of methylation in actin functions [12]. By the late 1970s, it was confirmed that only a single histidine residue in actin is *Nτ*-methylated, and the residue is located precisely at position 73 of the amino acid sequence [13]. However, it was only in 1987 that the presence of actin histidine methyltransferase in the myofibrillar fraction of rabbit muscle was shown for the first time [14]. The advent of recombinant DNA technology allowed better characterization of a partially purified rabbit enzyme by using nonmethylated recombinant actin which was heterologously expressed in *Escherichia coli* and a synthetic peptide corresponding to residues 69–77 of actin [15]. In addition, it was also proved that rabbit skeletal muscle is a source of two different histidine methyltransferases. The first of these enzymes was specific for actin, while the second one—carnosine *N*-methyltransferase—converts carnosine (β-alanyl-L-histidine) into anserine (β-alanyl-*Nπ*-methyl-L-histidine) dipeptides, which are abundantly present in mammalian skeletal muscle. The carnosine-methylating enzyme was later identified as the UPF0596 protein, in eukaryotes [16]. Finally, pioneering studies carried out in 2002, employing actin monomers in methylated or nonmethylated forms, revealed that the methylation of actin at histidine 73 (H73) may facilitate its polymerization [3]. However, since these results were based on a functional comparison of actin monomers isolated from two different species—*Saccharomyces cerevisiae* and cow—their interpretation was difficult and the biological significance of such modification was uncertain.

Only recently, a putative histone lysine methyltransferase, SETD3, has been identified as actin specific histidine *N*-methyltransferase, and shown to regulate cytoskeleton assembly and modulate smooth muscle contractility [17,18] (Figure 1). This finding encouraged the scientific community to conduct more systematic searches for novel protein histidine methyltransferases and their substrates. Indeed, it was recently found that METTL9 methyltransferase acts as a broad specificity enzyme, catalyzing the formation of the majority of *N*π-methylhistidine residues in the human proteome, including S100A9 and NDUFB3 proteins [19]. This was also confirmed by Lv and colleagues, who established that METTL9 recognizes an xHxH motif in substrate proteins [20], whereas proteomic studies indicated that the motif is mainly present in human proteins that are methylated at histidine residues [21]. Moreover, the human METTL18 enzyme was shown to *Nτ*-methylate histidine 245 in ribosomal protein RPL3 [22,23], and, thus, resembles its yeast homolog HPM1 protein [7,24]. Histidine methylation has now been found to be prevalent in human cells, involving hundreds of intracellular proteins, which implies that the human proteome may contain several unidentified protein histidine methyltransferases [21].

In this review, we discuss the current advances in research on the SETD3 protein that were stimulated by its identification as the first protein histidine *N*-methyltransferase in metazoans and the renewed interest in histidine methylation as an important mechanism regulating protein functions.

## 2. The Structural Features of SETD3

SETD3 has a core SET domain (Su(var)3-9, Enhancer-of-zeste (E(z)), and Trithorax (Trx)), which is found in various proteins. In *Drosophila melanogaster*, all these genes code for proteins engaged in posttranslational modifications of histone H3 and transcriptional regulation: (i) Su(var)3-9 encodes [histone H3]-lysine(9) *N*-methyltransferase (EC 2.1.1.355), (ii) E(z) encodes [histone H3]-lysine(27) *N*-trimethyltransferase (EC 2.1.1.356), and (iii) Trx encodes [histone H3]-lysine(4) *N*-methyltransferase (EC 2.1.1.354). The SET domain is typical for enzymes exhibiting methyltransferase activity, and, as indicated by the names of the above mentioned enzymes, the presence of this domain is often associated with methyltransferase activity on lysine residues within the protein substrate. Indeed, SETD3 was initially identified as histone lysine *N*-methyltransferase [25,26], although the enzyme was shown to function as an actin specific histidine *N*-methyltransferase [17,18]. Interestingly, a follow up study by Dai et al. [27] demonstrated that the substitution of histidine by methionine in the actin derived peptide increases its affinity for the SETD3 protein by 76-fold. On the other hand, the substitution of lysine with methionine at K27 and K36 residues was found in histone H3.3 [28,29]. At present, the oncogenic effects of these substitutions are primarily linked with the perturbation of proper lysine methylation [30]. However, the results of Dai et al. [27] suggest that SETD3 in vivo may act as a methionine methyltransferase.

### 2.1. Domain Architecture

The human SETD3 protein (NCBI Protein: NP_115609.2) consists of 594 amino acid residues and has a molecular weight of 67.26 kDa. In addition to the well characterized isoform 1, there are two isoforms containing 296 and 286 amino acids, respectively. The structural characteristics described hereafter refer to isoform 1.

SETD3 has a 250-residue long SET domain (residues 80–329) which ensures specific recognition of the actin derived peptide, and most probably, the actin molecule itself. This domain is larger than a typical SET domain due to the presence of an inserted region (residues 131–254), designated as iSET. The regions that are responsible for AdoMet binding are located within the SET domain (residues 105–106, 275–279, and 313). Structural studies conducted in recent years have revealed the actual interactions occurring between SETD3 and *S*-adenosyl-homocysteine (AdoHcy), which is a product of AdoMet demethylation [18,31], or sinefungin (SFG; adenosyl ornithine), which is an AdoMet analog lacking the ability to transfer a methyl group [32,33] and anticipated as a binding site of AdoMet.

**Figure 2:**
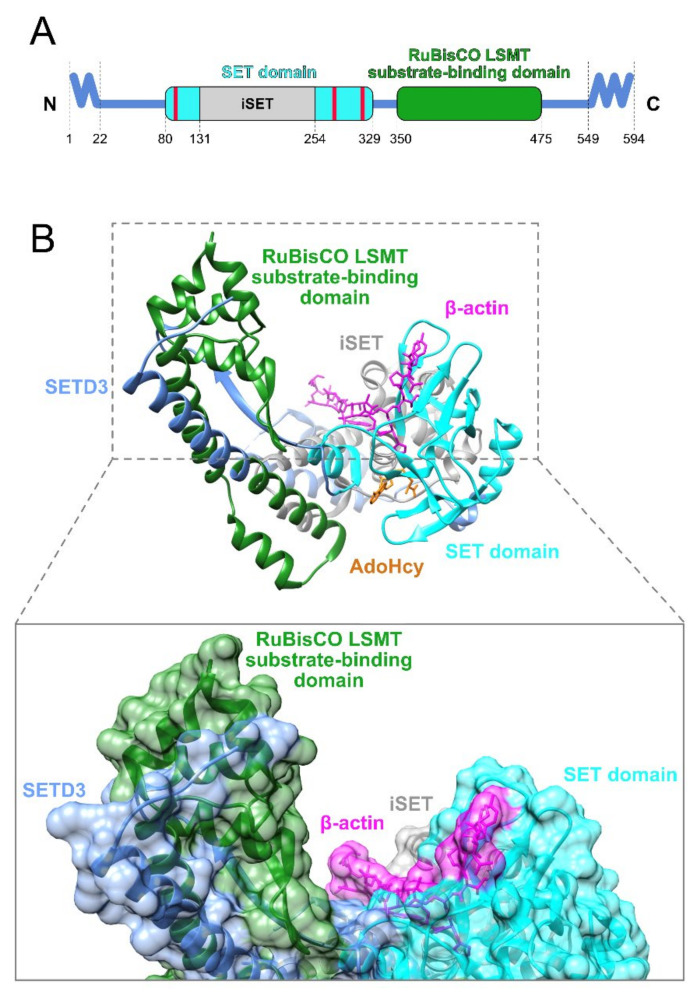
Structure of human SETD3. (**A**) Domain composition of SETD3. Waved lines correspond to the disordered regions at the N-terminal and C-terminal of the protein. Red bars indicate the localization of amino acid residues at which AdoMet binds to SETD3. Data were retrieved from the NCBI Protein database (accession number: XP_011535533.2, accessed on 30 July 2021). (**B**) Conformation of human SETD3 (residues 2–502) in complex with the peptide substrate derived from β-actin (residues 66–88) and close up view of the SETD3 substrate binding cleft with molecular surfaces. The image was created in UCSF Chimera 1.15 software utilizing the coordinates deposited in Protein Data Bank file 6ICV [36]. The color scheme of domains is common in Figure 2 and Figure 3.

The residues 350–475 of SETD3 are folded into a domain that structurally resembles the RuBisCO LSMT (large subunit methyltransferase) substrate binding domain [31]. In LSMT, the substrate binding domain interacts specifically with the RuBisCO large subunit [34,35]. Thus, it seems that the LSMT substrate binding domain present in the SETD3 protein may be involved in the recognition and binding of protein substrates, although experimental data supporting this hypothesis are scarce. The N-terminal and C-terminal regions (residues 1–22 and 549–594, respectively) of the SETD3 protein are considered to be disordered (Figure 2A).

Several single amino acid substitutions can significantly influence the catalytic activity and/or specificity of SETD3. For example, Guo et al. [31] reported that R215A and R316A reduced the affinity of protein histidine *N*-methyltransferase for the actin derived peptide substrate, and decreased the enzyme activity.

A similar effect of diminished affinity to the actin derived peptide and lower enzyme activity was also found to be triggered by N256A and N256V substitutions [31,32], although the lowest binding affinity was observed with N256D substitution [31]. This finding suggests that the presence of a negative charge at this position may have a detrimental effect on substrate binding. However, the mentioned substitutions allow SETD3 to bind the variants of actin derived peptides with amino acid substitutions within the target sequence, and catalyze the methylation of lysine or methionine, as indicated above [27]. A different substitution at the same amino acid residue (N256F), in combination with W274A substitution, was also shown to trigger protein lysine methyltransferase activity to an actin derived peptide variant containing lysine, instead of histidine, in the target sequence [27].

Wilkinson et al. [18] observed that Y313A substitution affected the activity of SETD3 protein histidine *N*-methyltransferase, while Y313F substitution, which only removed the hydroxyl group present in the ortho position on the benzene ring, strongly decreased the binding of protein histidine *N*-methyltransferase to the actin fragment, as well as the enzyme activity [31]. This implies that the hydroxyl group of Y313 is critical for the proper recognition of the substrate by the SETD3 protein, and its catalytic activity.

### 2.2. Structure

The 3D structures of SETD3 in complex with an unmethylated or methylated actin-derived peptides were successfully determined by applying the X-ray diffraction crystallography technique. Both structures were solved using crystals containing AdoHcy, which was added to the buffer to prevent methylation of a peptide substrate. AdoHcy is one of the products of this reaction and occupies the catalytic pocket of the enzyme, thus preventing the binding of AdoMet [18,31]. Another approach involves the use of SFG, which fits into the catalytic pocket as AdoMet but does not transfer the methyl group [32,33].

Guo et al. [31] reported that the approach of cocrystallizing the SETD3 protein with full length actin was unsuccessful, and the obtained structures contained the core region of SETD3 (residues 2–502) and the peptide substrate derived from β-actin (residues 66–88). Wilkinson et al. [18] and Dai et al. [32], on the other hand, used full length SETD3 and an actin peptide substrate (residues 66–80). In all these structures, the core region of SETD3 adopted a V shape, resembling the canonical SET domains found in Su(var)3-9, E(z), or Trx [37]. However, the SET domain of the SETD3 protein largely resembles the SET domain of LSMT [34], due to the presence of an inserted α-helical domain bisecting the SET domain, namely, iSET [31,38].

AdoHcy (and also most probably AdoMet) interacts with SETD3 in a cleft formed by the SET domain, which is additionally supported by a fragment of the iSET domain (Figure 2B). Its adenine ring is located between the side chain of E104 and the aromatic ring of F327. The AdoHcy N6 and N7 atoms are supported by hydrogen bonds formed with the main chain carbonyl and amide groups of H279, respectively, while its C8 atom forms a hydrogen bond with the hydroxyl group of Y313 [31]. The mode of interaction of AdoHcy with SETD3 is analogous to that observed in other SET containing enzymes, such as LSMT [34] and SETD6 [39].

The peptide substrate derived from β-actin interacts with SETD3 in a narrow cleft formed by the SET domain including the iSET region—in the same cleft where AdoHcy is located. However, the peptide substrate for histidine methylation is located at the lowest part of a wider cleft on the surface of SETD3. This spacious cleft might serve as an interaction site for larger unidentified protein substrates, together with the RuBisCO LSMT substrate binding domain (Figure 2B).

**Figure 3:**
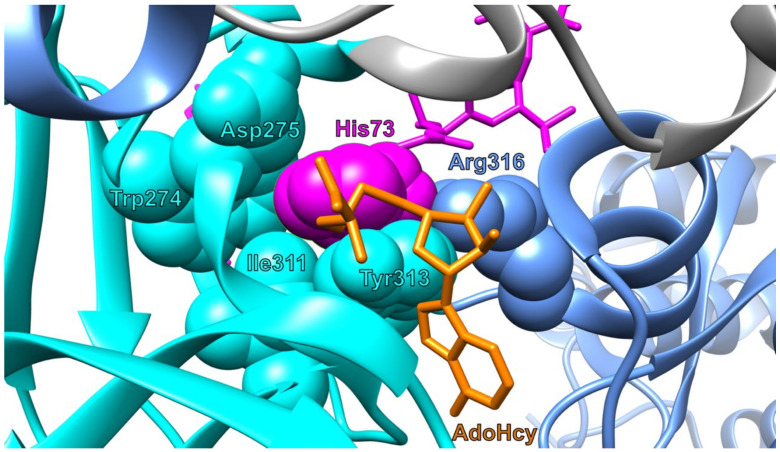
Amino acid residues of SETD3 that are important for proper recognition and alignment of H73 of β-actin to AdoMet prior to methylation. The image was created in UCSF Chimera 1.15 software utilizing the coordinates deposited in Protein Data Bank file 6ICV [36]. The color scheme of domains is common in Figure 2 and Figure 3.

The methylated H73 residue of β-actin fits into a hydrophobic pocket formed by W274, I311, and Y313 of SETD3 [31] (Figure 3). The imidazole ring of H73 is aligned parallel to the aromatic ring of tyrosine 313. Its orientation is determined by two hydrogen bonds—one formed between the N1 and N3 atoms of the imidazole ring and another between the guanidino group of R316 and the carbonyl group in the main chain of N275 [31]. According to a recent study, the substrate binding pocket of SETD3 is charged in a way that corresponds to the surface charge of the actin fragment fitting to it, which also contributes to the proper alignment of the substrate to the enzyme [33].

Interestingly, the β-actin derived peptide adopts a 3_10_ helix at its C-terminus only when H73 is methylated. However, the overall structure of the complex is very similar to that before methylation, which is confirmed by a root mean square deviation of 0.19 and 0.32 Å over protein and peptide Cα atoms, respectively [31].

SETD3 structural investigations support the notion that the enzyme is primarily a histidine *N*-methyltransferase [17,18], and not a lysine *N*-methyltransferase, as it was initially classified [25,26]. The key argument for this is that the substrate-binding site of the SETD3 protein fits very well to the β-actin peptide, but it might be too shallow for the stable binding of the aliphatic side chain of a lysine residue. On the other hand, the wide cleft present above the substrate-binding pocket may allow the interaction of SETD3 with other protein substrates.

It is worth noting, though, that substitutions of N256 in SETD3 to other amino acid residues influence the substrate-binding affinity and/or specificity. Importantly, in the case of structurally similar SET-domain-containing (SETD) enzymes, such as LSMT or SETD6, this position may contain a phenylalanine residue, which is responsible for enzyme interaction with the lysine side chain present in substrate proteins [31]. These findings substantiate the reclassification of SETD3 as a histidine *N*-methyltransferase.

### 2.3. Paralogs

The existence of SETD3 paralogs is still unknown. However, based on the amino acid sequence, it can be suggested that the SETD4 protein, with 40% similarity and 24% identity, may be considered as a potential paralog. SETD4 is a histone lysine *N*-methyltransferase (EC 2.1.1.364), which catalyzes the methylation of histones H3 and H4 at K4 and K30 residues, respectively. It was reported that this enzyme regulates cell proliferation, differentiation, inflammatory response, and heterochromatin formation [40]. The domain structure of SETD4 resembles that of SETD3. Although the amino acid sequence of SETD4 is shorter than that of SETD3 and contains only 440 residues, the SET domain consisting of 226 residues is in the central part of the protein (residues 48–273). The N-terminus of SETD4 is also disordered (residues 1–24), similar to SETD3.

**Figure 4:**
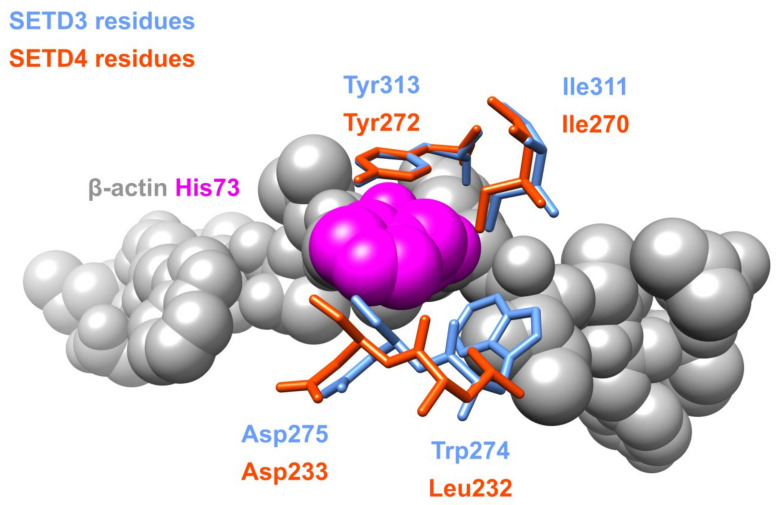
Structural alignment of SETD3 amino acid residues interacting with H73 of β-actin and conserved residues of SETD4. The image was created in UCSF Chimera 1.15 software utilizing the coordinates deposited in Protein Data Bank file 6ICV and the SETD4 structure predicted by AlphaFold [41] using UniProt Q9NVD3 record as an input. Structural alignment was calculated using the MatchMaker tool in UCSF Chimera 1.15 software [36].

In order to analyze the potential structural and functional convergence of SETD3 and SETD4, we predicted the structure of human SETD4 using the AlphaFold algorithm [41]. Interestingly, three out of the five residues participating in substrate binding in SETD3 (described below) are conserved in SETD4. Moreover, the Y313 residue, which ensures the appropriate alignment of the imidazole ring of histidine substrate in SETD3, is structurally conserved in SETD4 as Y272 (Figure 4). This may signify that SETD4 shows potential SETD3-like protein histidine *N*-methyltransferase activity, although no experimental evidence is available to confirm this hypothesis.

Notably, the overall fold of SETD3 is highly similar to that of RuBisCO LSMTs and SETD6, both of which are validated protein lysine methyltransferases. However, SETD3 has low sequence identity with RuBisCO LSMTs and SETD6 (24–25%) [31]. Therefore, these enzymes cannot be listed as closely related paralogs of SETD3, but it can be concluded that the fold of SETD3 is not unique.

## 3. The Biochemical Features of SETD3

**Figure 5:**
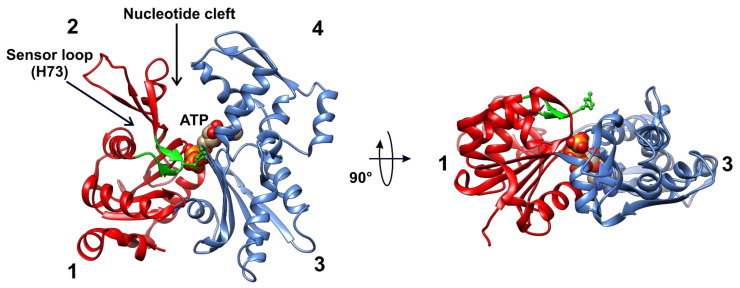
Structures of human β-actin. Ribbon representations of the structures of the actin monomer are shown in different projections. The actin molecule consists of small and large domains (red and blue, respectively), and each one is divided further into two subdomains: 1, 2, and 3, 4, respectively. ATP (or ADP) binds to the cleft between subdomains 2 and 4. The methyl-accepting H73 is located in a sensor loop spanning P70 to N78 (green). This residue is exposed to the surface of the actin monomer and seems to be easily accessible for SETD3. The model was prepared using UCSF Chimera [36] from the Protein Data Bank structures of β-actin (2BTF).

For many years, SETD methyltransferases were exclusively considered as enzymes responsible for the methylation of specific lysine residues at histone proteins and thereby for maintaining and altering the histone code [42]. Nevertheless, this viewpoint gradually changed as more number of nonhistone substrates for SETD methyltransferases were discovered [43]. Not surprisingly, SETD3 was initially thought as an enzyme that catalyzes the modification of histone H3 at K4 and K36 residues and regulates muscle cell differentiation in mice [26]. This was later confirmed by Chen and colleagues [44], who, however, also suggested that SETD3 might act on other nonhistone substrates in the cytoplasm, as the enzyme contains RuBisCO LSMT substrate-binding domain. Once the consensus on its role as a lysine-methylating enzyme began to take shape, SETD3 was identified as a long sought, actin specific histidine *N*-methyltransferase that catalyzes H73 methylation in the actin protein of metazoans [17,18] (cf. Figure 1 and Figure 5). This discovery was made by two independent research groups with their own dedicated research strategy. Studies performed in our laboratory [17] were based on the extensive purification of the native rat enzyme from leg muscles, using different chromatographic methods, and the subsequent molecular identification of the enzyme by tandem mass spectrometry. After two independent and slightly different rounds of purification, SETD3 methyltransferase was found as the only logical candidate for the enzyme. This discovery was then confirmed by generating recombinant homogenous rat and human SETD3 and determining their actin histidine-methylating activity. Finally, an analysis of SETD3 deficient *D. melanogaster* larvae and the human HAP1 knockout (KO) cell line proved that actin did not undergo histidine methylation in both the examined sources [17]. At the same time, Wilkinson and colleagues [18] analyzed previous evidence supporting the substrate specificity of SETD3 and questioned whether histones were appropriate substrates for this enzyme. To identify the proteins that are methylated by SETD3, recombinant human wild-type and catalytically inactive variants of SETD3 were prepared and incubated with a total cytoplasmic extract of human HT1080 cells in the presence of [^3^H]AdoMet. Autoradiography analysis revealed that the only detected band corresponded to a protein with a molecular weight of ≈42 kDa. Then, using mass spectrometry, the potential substrates were purified and identified. The most likely candidates were produced in *E. coli* and tested as SETD3 substrates in vitro. It was observed that only actin was methylated by the enzyme. The specific actin residues modified by SETD3 were identified by tandem mass spectrometry. Unexpectedly, no lysine methylation events were detected on the actin protein, and instead, the H73 residue was unambiguously identified as the sole target of SETD3 [18].

### 3.1. Substrate Specificity

#### 3.1.1. Actin

In vitro and in vivo experiments have proven that actin is the only known bona fide substrate of SETD3. There are three main isoforms of this protein—α, β, and γ—which differ only by a few amino acids at their N-terminus [45]. Under physiological conditions, actin exists as a 42-kDa monomeric globular protein (G-actin) that binds ATP and spontaneously polymerizes into relatively stable filaments (F-actin). The G-actin molecule consists of small and large domains, which are further subdivided into subdomains 1, 2, and 3, 4, respectively (Figure 5). The cleft between subdomains 2 and 4 is occupied by ATP or ADP. The methyl-accepting H73 residue is located in a sensor loop (P70 to N78), inserted between subdomains 1 and 2. The residue is exposed to the surface of the actin monomer and can thus be easily accessed by SETD3 (Figure 5).

The activity of SETD3 on actin has, so far, been studied using two different substrates: homogenous recombinant human β-actin produced in *E. coli* and an array of synthetic peptides of varying lengths, corresponding to the sensor loop of actin. Of note, full length recombinant actin monomers were purified from bacterial inclusion bodies in denaturing conditions and refolded into a nucleotide free state that represents a quasinative and nonphysiological form of this protein [17]. As actin requires eukaryotic chaperonins for correct folding, it cannot be produced in its native form in bacteria [46].

Radiochemical studies employing quasinative actin and [^3^H]AdoMet revealed the high affinity of human SETD3 toward both substrates with at least 60- and 300-fold lower K_M_ values (≈0.8 and ≈0.1 µM) than their intracellular concentrations, respectively [17]. The enzyme was also found to exhibit slow activity with a *k_cat_* value of about 0.7 min^−1^, which seems to be typical for methyltransferases acting on protein residues [47]. More interestingly, a comparison of the activity of SETD3 on either recombinant actin produced in *E. coli* or protein produced in *S. cerevisiae*, indicated that the enzyme catalyzed the methylation of only nucleotide free actin from bacteria. Thus, the yeast produced protein, which was nonmethylated due to the lack of SETD3 homolog in *S. cerevisiae* and expected to have a native conformation, could not serve as a substrate for SETD3 unless it was purified in the nucleotide free form [17]. Based on these results, it was interpreted that SETD3 may act on a specific form of actin monomers, plausibly nucleotide free actin, in a complex with one or more actin-binding proteins of unknown identity. This hypothesis is consistent with the current knowledge about SETD methyltransferases. Many of these enzymes form complexes with different proteins, and those interactions are important for their catalytic activity and substrate specificity [42].

Structural and biochemical studies using actin peptides have provided valuable data on the substrate binding and catalytic mechanism of SETD3. It was reported that actin-derived peptides bind in a long groove at the surface of the SET domain of the enzyme, with the H73 residue located within the active site pocket [31,32] (Figure 2B). The affinity of binding was found to increase with increasing peptide length (K_M_ = 8.7 mM and 21 µM for 9-residue and 15-residue peptide, respectively) [17,32]. However, those peptides containing H73M or H73K mutation were still methylated at position 73 [27,48], which suggests that peptide recognition is mainly sequence specific, rather than targeted residue (histidine)-specific, and, thus, SETD3 can target proteins other than actin, at residues other than histidine. Moreover, the substrate specificity of SETD3 can be altered by engineering critical amino acids in its active site. Only recently, a mutated variant of SETD3 harboring N256F and W274A substitutions was shown to exhibit a 13-fold higher affinity for lysine over histidine [48].

#### 3.1.2. Other Substrates

Studies on SETD3 employing peptide substrates allowed insight into the structural basis of H73 methylation and the catalytic reaction. However, it should be noted that this peptide based approach is a simplification. In fact, such a research model explores only local interactions occurring within the catalytic domain of SETD3, and ignores the entire spectrum of interactions occurring between the enzyme, particularly its RuBisCO LSMT substrate-binding domain, and the protein substrate. Thus, it is not unwise to speculate that RuBisCO LSMT is mainly responsible for controlling the substrate specificity of SETD3, and the enzyme may accept more substrates than only actin. Previous reports based on radiochemical assays have also shown that mammalian core histones, particularly histone H3, were the substrates for SETD3 [25,26,44]. However, such an activity of the enzyme was not detected in other works [18]. This apparent discrepancy might be explained by different sources of nucleosomes used in enzymatic assays. It seems that SETD3 may act on the isolated native nucleosomes [26,44], but not on recombinant ones [18] or free histone octamers [44]. If true, the targeted amino acid residue(s) must be verified, as data supporting H3 methylation at K4 and K36 sites [25,26] are unconvincing [18]. Finally, Cohn and coworkers [49] have shown that human SETD3 interacts with about 170 different intracellular proteins, including actin, which suggests that there may be many other substrates for this enzyme in mammalian cells.

#### 3.1.3. Inhibitors

Although the M73-containing peptide is a poor substrate for SETD3, it has been found to exhibit strong affinity to the enzyme and inhibit the methylation of the H73 peptide. Based on this observation, actin based peptidomimetics that act as effective substrate competitive inhibitors of human SETD3 were developed [50]. These are 16-residue-long analogs of the actin peptide (66–81), in which the H73 residue is substituted by a simple natural or non-natural amino acid. Among an array of tested peptide analogs, selenomethionine-containing actin peptide was identified as the most potent inhibitor of the human enzyme, with an IC_50_ value of 0.16 µM.

### 3.2. Reaction Mechanism

**Figure 6:**
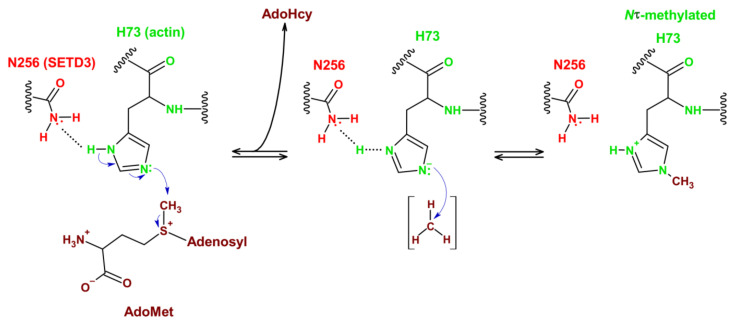
Plausible mechanisms of actin histidine *Nτ*-methylation by SETD3. The enzyme catalyzes the methylation of *N*τ nitrogen atom of H73 residue in actin. The methyl group of AdoMet can be transferred only to the deprotonated nitrogen atom. Since each of the two nitrogen atoms of the imidazole ring can hypothetically be protonated, the side chain of N256 residue of SETD3 stabilizes the *N*π nitrogen atom of H73 in the protonated form [32]. Consequently, it enhances the nucleophilicity of the lone pair of electrons present at the deprotonated *N*τ nitrogen that may then attack the methyl group of AdoMet.

The imidazole ring of the histidine residue contains two nitrogen atoms at different positions: 1 (π) and 3 (τ) (Figure 1). These nitrogen atoms can be protonated, resulting in the formation of an imidazolium cation, and each of them can subsequently release a proton to produce a different imidazole tautomer (Figure 1). Both fully protonated and tautomeric forms of the imidazole side chain are believed to be present at physiological pH ≈ 7 in proteins [51]. Similar to other AdoMet dependent methyltransferases, SETD3 appears to catalyze a conventional S*_N_*2 methylation reaction, in which the methyl group of AdoMet is transferred to the deprotonated *Nτ* nitrogen [32] (Figure 6). To facilitate this reaction, the side chain of N256 of the enzyme stabilizes the *Nπ* nitrogen of the substrate H73 residue in the protonated form, whereas the lone electron pair present at the deprotonated *Nτ* attacks the methyl group of AdoMet. This model of SETD3 catalysis is consistent with the findings that (i) the enzyme has an optimum pH of 7 and above for H73 methylation (pKa of 6.5 for histidine imidazole) [31], whereas a K73-containing actin peptide is readily methylated only at a pH above 9.5 (pKa of 10.5 for lysine side chain) [52], and (ii) the substitution of N256 by amino acids that cannot form a hydrogen bond with the protonated Nπ nitrogen results in a reduction or complete loss of SETD3 activity toward H73 residue [48].

### 3.3. Tissue Distribution and Intracellular Localization

**Figure 7:**
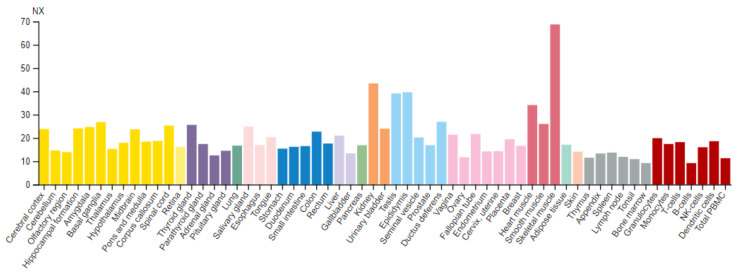
SETD3 expression in human tissues. RNA data were obtained from the Human Protein Atlas (HPA; https://www.proteinatlas.org, accessed on 29 July 2021) and show consensus normalized expression levels, determined by combining the data from three transcriptomic datasets (HPA, Genotype-Tissue Expression, and FANTOM5) [53]. Color coding is based on tissue groups, each consisting of tissues with common functional features.

The SETD3 protein or its orthologs are present in most of the eukaryotic organisms, including vertebrates (*Homo sapiens*, *Mus musculus*), plants (*Vitis vinifera*), insects (*Onthophagus taurus, D. melanogaster*), and fungi (but not in *S.* *cerevisiae*) [17]. The profile of SETD3 expression in humans shows relatively low tissue specificity (Figure 7).

The SETD3 mRNA is ubiquitously expressed at a similar basal level in most examined tissues, with the noticeable exception of the skeletal muscle, kidneys, and testes. The widespread expression of the enzyme is consistent with its function as an actin histidine methyltransferase because actin proteins are found in virtually all cells. The expression of STED3 has been shown to be highest in muscles, which is not surprising given the fact that muscle fibers are abundant in actin filaments [54]. This finding is also in good agreement with the enzymatic data, indicating the skeletal muscle as a rich source of actin specific histidine methyltransferase [14]. On the other hand, the augmented expression of SETD3 in kidneys and testes is more puzzling. It could be hypothesized that increased SETD3 expression is related to actin, which is an important protein in these two organs. It is well known that the dynamic remodeling of the actin cytoskeleton is important for efficient mammalian spermatogenesis [55] and for maintaining the functional structure of renal podocytes [56]. However, it cannot be ruled out that higher SETD3 expression in kidneys and testes is due to the role of this enzyme in the methylation of substrates other than actin. The intracellular localization of SETD3 is not well defined yet. Initial studies proposed that the enzyme is localized in the nucleus [26,49]. However, the enzyme was clearly detected in the cytosol [57] and mitochondria of mammalian cells [53].

## 4. The Cellular Features of SETD3

### 4.1. Biological Effect of Actin Methylation by SETD3

It is now clear that SETD3 is mainly actin histidine methyltransferase, and actin is its most important physiological substrate. However, the exact role of actin methylation is not clear.

#### 4.1.1. Polymerization of Actin

**Figure 8:**
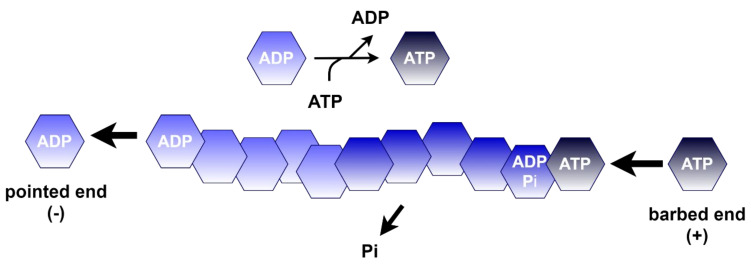
Scheme of nucleotide exchange under steady state during actin polymerization. During the steady state phase of polymerization, ADP–actin complexes dissociate from the pointed end (-) of the filamentous actin. This is followed by nucleotide exchange (from ADP to ATP) and, consequently, ATP–actin associates mainly at the barbed end (+). ATP hydrolysis allows the translocation of subunits between the ends of the filament [45]. SETD3 is found to promote actin polymerization through H73 methylation [18].

The presence of actin filaments ensures the stable structure and internal movement of cells [58]. β-Actin is the main cytoskeleton protein [59]. Actin polymerization involves nucleation, elongation, and steady state phases [60], and closely correlates with the concentration of actin monomers. Monomers are stabilized by ATP or ADP binding, but neither dimer nor trimer is stable and are therefore present in an extremely low concentration in the intracellular environment. The oligomer is only partially protected by the addition of four subunits [58]. Actin polymerization is followed by the hydrolysis of ATP to ADP and phosphate [61], which results in the polarity of actin filaments. The pointed end (-) of the actin filament is disassembled more freely, ensuring the presence of subunits that are added at the opposite, barbed end (+). Thus, there exists a balance between filament shortening and elongation [45] (Figure 8). Furthermore, it is well established that the remodeling of filaments requires many different proteins, including myosin, cofilin, profilin, capping proteins, or the Arp2/3 complex. These proteins, for example, promote phosphate dissociation in F-actin or nucleotide exchange in its G form [58]. Methylation of the actin protein at H73 also seems to be implicated in its remodeling, indicating the biological importance of the SETD3 activity.

#### 4.1.2. Effect of Actin H73 Methylation

Studies performed in the last 50 years attempted to elucidate the importance of H73 methylation in actin. Initially, it was indicated that such methylation is neither obligatory nor necessary for the proper functioning of actin [12,62]. Furthermore, actin with H73 substitutions by arginine or tyrosine residues was shown to polymerize as effectively as the nonmutated protein [62]. By contrast, a recent study revealed that lack of actin methylation affected the stability of actin monomers in SETD3-KO cells. The instability of actin monomers might lead to the accelerated depolymerization of actin fibers, and a loss of cytoskeleton integrity [17]. However, Wilkinson [18] reported that the methylation of actin promotes its polymerization, but without any impact on depolymerization. Thus, further research is needed to better understand the effect of H73 methylation on the stability of actin filaments.

### 4.2. The Cellular Roles of SETD3 and Association with Signaling Pathways

SETD3 is located mainly in the cytosol, and β-actin is the only cytosolic substrate described for this enzyme so far. However, it seems likely that the enzyme also acts on other substrates. Based on a proteomic approach, it was identified that more than 150 proteins, including cytoskeleton and signal proteins, receptors, hydrolases, and transcription factors, interact with SETD3 [49]. Therefore, it has been postulated that the enzyme may play a role in various biological processes, including myocyte differentiation [26], maintaining cytoskeleton integrity [17], cell cycle regulation and apoptosis [25], response to hypoxic conditions [49], carcinogenesis [44], and enterovirus (EV) pathogenesis [63].

#### The Functions of Cytosolic SETD3

In addition to its contribution to maintaining cytoskeleton integrity, SETD3 was shown to be involved in the pathogenesis of some EVs [63]. Although several studies have been performed on EVs, the precise mechanisms promoting their replication in target cells are unknown. It was shown that the formation of viral particles was diminished in SETD3-KO cells compared to wild type cells, which indicates that the enzyme supports the replication of viral genomes [63]. More interestingly, the level of replication in cells expressing the catalytically inactive SETD3 mutant was found to be in the control range, suggesting that the methyltransferase activity is not pivotal to viral multiplication. On the other hand, SETD3 was identified to strongly interact with viral protease 2A, and this interaction depends on the presence of both SET and RuBisCO LSMT domains in the enzyme structure [63]. It is well known that viral protease 2A, in combination with protease 3C, is essential for the completion of the EV life cycle. Neither the cleavage of the polyprotein into structural proteins during the replication cycle of EVs, nor the cleavage of the host protein, can occur without the activity of these proteases [64]. Moreover, they are implicated as possibly involved in suppressing stress and antiviral IFN-α/β responses [65]. These findings shed new light on the biological significance of the SETD3 protein, and highlight it as crucial for the successful reproduction of some EVs.

### 4.3. Other Postulated Functions of the SETD3 Protein 

Attempts have been made to explore the potential role of SETD3 in carcinogenesis [44,66,67,68]. The available information collectively suggests the importance of SETD3 in the development and progression of cancer [44,49], as discussed in the next section.

The other assumed functions of SETD3, including myocyte differentiation, response to hypoxia, and cell cycle regulation, are attributed to the implied histone methylation by this enzyme or its nuclear localization.

As the first proposed activity of SETD3 was H3 methylation, its role in the epigenetic regulation of chromatin was also considered [25,26]. The abundant presence of SETD3 in muscles has been indicated to induce myocyte differentiation. In C2C12 or H9c2 cells, the overexpression of SETD3 activated the transcription of MCK, Myf6, and myogenin genes, which code for proteins involved in myocyte differentiation, whereas SETD3 knockdown was found to inhibit the differentiation of muscle cells. Nevertheless, the transcriptional activation of muscle-related genes by SETD3 needs to be confirmed by further research [26].

It has also been reported that the transcription factor FoxM1 is bound and methylated by SETD3 in vitro [49]. FoxM1 is crucial for the self renewal and proliferation of cells [69]. This is in line with the observation that SETD3 strongly interacted with FoxM1 at chromatin in normoxia, but its association with FoxM1 was weaker under hypoxic conditions. Furthermore, SETD3, along with FoxM1, regulated the expression of VEGF. The dissociation of both SETD3 and FoxM1 from the VEGF promoter was suggested to increase VEGF expression and promote angiogenesis in hypoxic conditions [49].

The functions of SETD3 reported by various studies are summarized in Table 1. Although literature data point out that SETD3 is associated with several signaling pathways, this protein has relatively recently been recognized to act mainly as actin histidine methyltransferase. This implies that its significance in biological processes is largely unexplored and warrants more studies in the future.

## 5. The Role of SETD3 in Diseases

The knowledge about the role of SETD3 in the pathogenesis of various diseases remains limited. However, since the discovery and molecular characterization of SETD3 as a histone H3 methyltransferase [25,26,44] and further studies redefining its biological role as an actin H73 methyltransferase [17,18], a growing body of evidence has suggested that the protein may play an ambiguous role in diseases, especially cancer or other abnormalities. Therefore, the following part of the paper summarizes the most current knowledge regarding the potential involvement of the SETD3 protein in pathogenesis, as well as its role as a biomarker in various diseases.

### 5.1. Cancer

Although the precise role of SETD3 in carcinogenesis is still unclear, available data confirm that the protein might act either as a cancer suppressor or as an oncogenesis-promoting factor. Interestingly, the role of SETD3 varies in different abnormalities and is therefore difficult to comprehend. It was previously shown that an SET-domain-lacking fragment of the SETD3 gene translocated to the immunoglobulin lambda light chain *locus* in B-cell lymphomas [44], which resulted in the disruption of the SETD3 gene and appearance of a shorter form of the SETD3 protein lacking the SET domain. Unexpectedly, this form of the protein accumulated in cancer cells, where the wild type could not. The truncated SETD3 was proposed to act as a dominant negative mutant promoting oncogenesis [44]. Nevertheless, the exact mechanism underlying the oncogenic effect resulting from the overexpression of the short form of SETD3 in lymphoma remains unknown.

The level of the SETD3 protein was observed to fluctuate during the cell cycle [57]. Specifically, it was highest in the S phase, but declined during the progression to the M phase. Such dynamic cell cycle dependent regulation of expression implicates a potential role for SETD3 in carcinogenesis. Indeed, the level of SETD3 was shown to be elevated in hepatocellular carcinoma (HCC) [57]. Two hypothetical mechanisms have been proposed for the decreased degradation of SETD3. The first one involves the mutational burden on the β-isoform of the FBXW7β tumor suppressor protein, which is required for the ubiquitination and proteolysis of SETD3 [57]. On the other hand, a couple of Cdc4 phosphodegrons (CPDs) were identified in the SETD3 sequence, and one of them, CPD1, was shown to be phosphorylated specifically by GSK3β. Not surprisingly, either a decrease in the activity of FBXW7β or GSK3β or mutations within the CPD1 region reduced the extent of degradation of SETD3 [57]. Moreover, it was recently reported that SETD3 is a poor prognostic biomarker in HCC patients [67] and patients with a high level of the protein had lower rates of recurrence free survival and overall survival after surgery. In addition, in vitro and in vivo studies revealed that SETD3 promoted the progression of HCC [57]. The use of SETD3 targeted shRNA resulted in the depletion of the protein and significantly inhibited the variability and colony formation of HCC cells [57]. Similar results were observed with the use of a xenograft tumor model, where the application of shSETD3 resulted in a decreased volume and weight of the abnormal tissues [57]. Surprisingly, the SETD3 protein inhibited metastasis in HCC cells. In vitro studies performed with Hep3B and SK-Hep-1 cell lines showed that SETD3 knockdown led to increased migration and invasion [67]. Furthermore, the SETD3-deficient SK-Hep-1 cells exhibited higher metastatic activity in the mice model than cells containing the functional gene [67]. In addition to promoting metastasis, the SETD3 protein was shown to regulate the expression of serine/threonine-protein kinase DCLK1 by DNA methylation. However, the exact role of SETD3 in DNA methylation remains to be investigated [67], while its DNA-methylating activity has never been described before.

It was recently reported that circRNA transcribed from SETD3 gene exons 2–6 was downregulated in HCC, and the level of the circSETD3 transcript correlated with tumor size and the malignant differentiation of HCC [70]. CircSETD3 is postulated to act as an miRNA sponge that downregulates the level of miR-421, an essential promoter of HCC. Intriguingly, the latest report on the role of circSETD in nasopharyngeal carcinoma revealed the opposite function of circSETD, and indicated that the transcript seems to promote the migration and invasiveness of nasopharyngeal carcinoma [71] by attenuating miR-615-5p and miR-1538. This, in turn, results in the upregulation of MAPRE1 expression and inhibition of α-tubulin acetylation [71]. Thus, the actual role of circSETD3 in carcinogenesis is unclear.

The role of SETD3 in breast cancer is largely determined by the expression of hormone receptors and the mutational status of the p53 protein. In triple negative breast cancer patients with a mutational burden within the p53 protein, the higher level of SETD3 protein was found to correlate with poor prognosis [68]. By contrast, in patients with estrogen receptor positive breast cancer, a higher level of SETD3 correlated with better clinical outcomes [68]. The SETD3 protein has been shown to regulate the expression of various genes associated with cancer progression, including FOXM1, ACTB, ASMA, ACTG, FSCN, and FBXW7. However, the regulation by SETD3 seems to be cell specific [68], and thus, it is difficult to decipher the role and mechanism of this protein.

The SETD3 protein was also implicated in the resistance of cervical cancer (CC) to radiotherapy [72]. With the use of the radioresistant SiHa cell line and a parental cell line lacking radioresistance, it was demonstrated that the level of the SETD3 protein negatively correlated with radioresistance, and its expression was downregulated in radiotherapy-resistant SiHa cells. Analysis of clinical samples from radiotherapy prone and resistant patients revealed comparable results [72]. The finding that SETD3 knockdown decreased the rate of cell death, DNA damage, and apoptosis raised a question regarding the mechanism involved in the protective effect of the SETD3 protein. The elevated level of this protein in CC was associated with decreased expression of KLC4, which was previously shown to participate in cell death by regulating DNA damage response in lung cancer cell lines [73]. However, additional studies are required for further clarification of the function of SETD3 in CC.

The SETD3 protein has been recently proven to act as a regulator of cell apoptosis [74] in colon cancer. Its higher expression was positively correlated with the rate of programmed cell death following doxorubicin treatment. A total of 215 proteins have been identified to interact with the overexpressed SETD3 protein, among which some are linked to RNA metabolism. However, the role of SETD3 in RNA metabolism remains to be investigated [74]. Interestingly, it was also shown that apoptosis was maintained only by the wild type SETD3 protein, while the substitution of tyrosine 313 to alanine (Y313A) attenuated the effect of the protein on the process. This suggests that the methylating activity of SETD3 might be crucial in the regulation of apoptosis [74]. SETD3 was also found to act as a positive regulator of the p53 protein, although it did not directly interact with or methylate the p53 protein [74].

The SETD3 protein may act as a prognostic biomarker in cancer. It was proposed that SETD3, along with the *N*-lysine methyltransferase SMYD2 and bifunctional lysine specific demethylase and histidyl-hydroxylase NO66, can be helpful in the diagnosis and prognosis of renal cell tumors [66]. Furthermore, clinical data proved that the downregulation of those proteins correlated with shorter disease specific and disease free survival [66]. Similarly, among different methyltransferases, the SETD3 protein was identified to be a key player in the progression of bladder cancer [66]. Nevertheless, the significance of the protein in this particular cancer has not been investigated so far and needs to be studied in the future. The SETD3 protein also seems to have a prognostic value in clear cell ovarian carcinoma [75].

The role of the SETD3 protein in oncogenesis is ambiguous because it may act as an oncoprotein and increase the effectiveness of anticancer therapies (i.e., radiotherapy or doxorubicin treatment). SETD3 might also be helpful to stratify patients according to clinical prognosis. However, additional studies should be performed to obtain more detailed data on the role(s) of SETD3 in the development of various malignancies, their progression, and invasiveness. Several studies published so far have focused on the role of the SETD3 protein in cancer, while only a few have addressed the potential involvement of this protein in other pathologies.

### 5.2. Other Diseases

As mentioned in Section 4.3, the SETD3 protein has been shown to be involved in the transcriptional regulation of VEGF expression under normoxia and hypoxia [49]. Under hypoxic conditions, the attenuated interaction of the SETD3-FoxM1 complex and promotion of the VEGF expression may result in the onset of hypoxic pulmonary hypertension [76]. On the other hand, overexpression of the SETD3 protein limits VEGF expression and HIF-1 activation and, thus, protects against hypoxic pulmonary hypertension [76].

It was recently shown that the SETD3 protein might be involved in the progression of autoimmune diseases, including systemic lupus erythematosus (SLE) [77]. The disease is associated with an elevated level of CXCR5 in CD4^+^ follicular helper T cells [77]. CXCR5 promotes the migration and interaction of T cells with B cells which, in turn, results in the formation of plasma cells through the interaction of PD-1 with its ligands (PD-1L and PD-2L) and production of autoantibodies. The SETD3 protein was elevated in the SLE CD4^+^ cells, and its level correlated with a higher expression of CXCR5 [77].

The SETD3 protein also has a protective effect on ischemia–reperfusion (I/R)-induced brain injury [78]. The level of SETD3 was found to be positively correlated with neuronal survival. The neuroprotective role of the protein was proposed to be related to the actin histidine-methylating activity and regulation of F-actin polymerization [78]. Physiologically, SETD3 expression was downregulated by the activity of PTEN phosphatase as a result of I/R-induced injury. In addition, the downregulation of SETD3 expression results in an increased level of reactive oxygen species, decreased mitochondrial membrane potential, and ATP production [78]. However, further studies are required to understand the mechanism underlying the complex crosstalk between the activity of PTEN phosphatase and the SETD3 protein in neurons.

Recently, it was reported that the actin histidine-methylating activity of the SETD3 protein plays a significant role in dystocia (delayed parturition) [18]. It was reported that the litter sizes of double mutated (*Setd3*^−/−^) mice were smaller than those of the wild type mice or mice with one functional allele. Nevertheless, this observation was inconsistent with the lack of anatomical abnormalities within the pelvis, and so the association of SETD3 with secondary dystocia was excluded [18]. A relationship between H73 methylation and uterine smooth muscle contraction was also proposed and verified experimentally. It was noted that the depletion of the SETD3 protein and actin H73 methylation resulted in a decreased signal induced contraction of primary human myometrial cells, while the intrinsic contractions were not affected [18]. Moreover, contractions induced by oxytocin and endothelin-1 were restored only by the catalytically active SETD3 protein but not by its mutated inactive form. All these data support the hypothesis that actin H73 methylation influences the signal induced contraction of smooth muscles [18].

The SETD3 protein was also shown to be involved in enteroviral infections [63]. Employing two human EVs—rhinovirus C15 (RV-C15) and EV-D68—SETD3 was selected as a hypothetical host factor essential for the infectiousness of EVs. The potential contribution of SETD3 in the pathogenesis of EVs is described in Section 4. An in vivo study indicated that SETD3 deficient (*Setd3^−/−^*) mice were viable and showed no symptoms of viral infection [63]. In the context of viral infections, the region encoding the SETD3 protein was recently shown to be an integration site in the precancerous human papillomavirus infections [79]. While only two reports are currently available regarding the importance of the SETD3 protein in viral contagiousness, it is extremely important, taking into account the current pandemic status, to investigate the role of host proteins in the progression of viral infections.

## 6. Outlook

Although studies have established that SETD3 is the long sought, actin specific histidine *N*-methyltransferase, the biochemical properties of this protein as well as the cellular processes it regulates are yet to be understood in detail. For instance, the crystal structure of the SETD3–actin complex has not been deciphered and attempts made so far to crystalize the complex were unsuccessful [31]. A possible explanation for this failure could be that the actual physiological form of actin bound and subsequently methylated by SETD3 is not known, and whether the substrate is F-actin, G-actin, or, perhaps, G-actin in a complex with unidentified protein(s) should be verified. However, data collected from experiments involving the purification of native SETD3 showed that the enzyme is tightly bound to myofibrils, suggesting that it forms a relatively stable complex with myofibrillar proteins [14,17].

Further work is needed to explain the functions of SETD3 methyltransferase in the cell nucleus. One may hypothesize that nuclear SETD3 exhibits different substrate specificity and targets histone H3, as has been previously shown for isolated human nucleosomes [44]. Intriguingly, avian histones were reported to undergo *N*τ–methylation at histidine residues [80], and so it would be interesting to verify whether SETD3 might be responsible for such modification. If true, SETD3 would be recognized as another dual specificity protein methyltransferase whose target activity depends on its interaction with a specific (non)substrate protein(s) [81,82]. Alternatively, the enzyme might work as a scaffold protein, facilitating the formation of a yet unknown protein complex, similar to that observed in the case of enteroviral protease 2A [63].

The regulation of SETD3 activity is another topic that remains to be investigated. All studies to date have focused only on mammalian SETD3. However, the enzyme is prevalent in multicellular eukaryotes. Thus, it would be of considerable interest to analyze the orthologs from more evolutionarily distant species, particularly in the plant kingdom. It is still unclear whether SETD3 catalyzes the methylation of histidine residues in plant proteins, and if so, what would be the physiological importance of SETD3 in plant species.

In conclusion, at the current research stage, our knowledge of the SETD3 protein seems to be in its infancy. Although a lot is known about the structure of SETD3 and the mechanism of actin H73 methylation, the understanding of the physiological importance of the enzyme is still very limited. Future research will need to address the above questions in more detail in order to gain in depth knowledge about SETD3.

## Figures and Tables

**Table 1:** Summary of the most important reported functions of SETD3 and its association with cell signaling pathways.

Process	SETD3 Activity	Proposed Functions	References
Actin polymerization	Methylation of H73 in β-actin	Stabilization of actin monomers	[17,18]
		Promoting actin polymerization	
Enterovirus pathogenesis	Unknown	Supporting viral genome replication	[63]
		Interaction with viral protease 2A	
Epigenetic regulation of chromatin	Methylation of core histones, plausibly H3	Regulation of gene expression	[25,26,44]
Response to hypoxia conditions	Methylation of FoxM1	Increasing expression of VEGF and promotion of angiogenesis	[49]
Carcinogenesis	Unknown	Regulation of cancer development and progression	[44,49,57,66,67,68,70,71,72,73,74,75]

## Data Availability

Not applicable.

## References

[1] Walsh C.T., Garneau-Tsodikova S., Gatto G.J. (2005). Protein posttranslational modifications: The chemistry of proteome diversifications. Angew. Chem. Int. Ed. Engl..

[2] Clarke S.G. (2013). Protein methylation at the surface and buried deep: Thinking outside the histone box. Trends Biochem. Sci..

[3] Nyman T., Schüler H., Korenbaum E., Schutt C.E., Karlsson R., Lindberg U. (2002). The role of MeH73 in actin polymerization and ATP hydrolysis. J. Mol. Biol..

[4] Raftery M.J., Harrison C.A., Alewood P., Jones A., Geczy C.L. (1996). Isolation of the murine S100 protein MRP14 (14 kDa migration-inhibitory-factor-related protein) from activated spleen cells: Characterization of posttranslational modifications and zinc binding. Biochem. J..

[5] Elzinga M., Collins J.H. (1977). Amino acid sequence of a myosin fragment that contains SH-1, SH-2, and Ntau-methylhistidine. Proc. Natl. Acad. Sci. USA.

[6] Meyer H.E., Mayr G.W. (1987). N pi-methylhistidine in myosin-light-chain kinase. Biol. Chem. Hoppe-Seyler.

[7] Webb K.J., Zurita-Lopez C.I., Al-Hadid Q., Laganowsky A., Young B.D., Lipson R.S., Souda P., Faull K.F., Whitelegge J.P., Clarke S.G. (2010). A novel 3-methylhistidine modification of yeast ribosomal protein Rpl3 is dependent upon the YIL110W methyltransferase. J. Biol. Chem..

[8] MacTaggart B., Kashina A. (2021). Posttranslational modifications of the cytoskeleton. Cytoskeleton.

[9] Johnson P., Harris C.I., Perry S.V. (1967). 3-methylhistidine in actin and other muscle proteins. Biochem. J..

[10] Asatoor A.M., Armstrong M.D. (1967). 3-methylhistidine, a component of actin. Biochem. Biophys. Res. Commun..

[11] Elzinga M. (1971). Amino acid sequence aroung 3-methylhistidine in rabbit skeletal muscle actin. Biochemistry.

[12] Johnson P., Perry S.V. (1970). Biological activity and the 3-methylhistidine content of actin and myosin. Biochem. J..

[13] Elzinga M., Collins J.H., Kuehl W.M., Adelstein R.S. (1973). Complete amino-acid sequence of actin of rabbit skeletal muscle. Proc. Natl. Acad. Sci. USA.

[14] Vijayasarathy C., Rao B.S. (1987). Partial purification and characterisation of S-adenosylmethionine:protein-histidine *N*-methyltransferase from rabbit skeletal muscle. Biochim. Biophys. Acta.

[15] Raghavan M., Lindberg U., Schutt C. (1992). The use of alternative substrates in the characterization of actin-methylating and carnosine-methylating enzymes. Eur. J. Biochem..

[16] Drozak J., Piecuch M., Poleszak O., Kozlowski P., Chrobok L., Baelde H.J., de Heer E. (2015). UPF0586 Protein C9orf41 Homolog Is Anserine-producing Methyltransferase. J. Biol. Chem..

[17] Kwiatkowski S., Seliga A.K., Vertommen D., Terreri M., Ishikawa T., Grabowska I., Tiebe M., Teleman A.A., Jagielski A.K., Veiga-da-Cunha M. (2018). SETD3 protein is the actin-specific histidine *N*-methyltransferase. eLife.

[18] Wilkinson A.W., Diep J., Dai S., Liu S., Ooi Y.S., Song D., Li T.M., Horton J.R., Zhang X., Liu C. (2019). SETD3 is an actin histidine methyltransferase that prevents primary dystocia. Nature.

[19] Davydova E., Shimazu T., Schuhmacher M.K., Jakobsson M.E., Willemen H.L.D.M., Liu T., Moen A., Ho A.Y.Y., Małecki J., Schroer L. (2021). The methyltransferase METTL9 mediates pervasive 1-methylhistidine modification in mammalian proteomes. Nat. Commun..

[20] Lv M., Cao D., Zhang L., Hu C., Li S., Zhang P., Zhu L., Yi X., Li C., Yang A. (2021). METTL9 mediated N1-histidine methylation of zinc transporters is required for tumor growth. Protein Cell.

[21] Kapell S., Jakobsson M.E. (2021). Large-scale identification of protein histidine methylation in human cells. NAR Genom. Bioinform..

[22] Małecki J.M., Odonohue M.F., Kim Y., Jakobsson M.E., Gessa L., Pinto R., Wu J., Davydova E., Moen A., Olsen J.V. (2021). Human METTL18 is a histidine-specific methyltransferase that targets RPL3 and affects ribosome biogenesis and function. Nucleic Acids Res..

[23] Matsuura-Suzuki E., Shimazu T., Takahashi M., Kotoshiba K., Suzuki T., Kashiwagi K., Sohtome Y., Akakabe M., Sodeoka M., Dohmae N. (2021). METTL18-mediated histidine methylation on RPL3 modulates translation elongation for proteostasis maintenance. bioRxiv.

[24] Al-Hadid Q., Roy K., Chanfreau G., Clarke S.G. (2016). Methylation of yeast ribosomal protein Rpl3 promotes translational elongation fidelity. RNA.

[25] Kim D.W., Kim K.B., Kim J.Y., Seo S.B. (2011). Characterization of a novel histone H3K36 methyltransferase setd3 in zebrafish. Biosci. Biotechnol. Biochem..

[26] Eom G.H., Kim K.B., Kim J.H., Kim J.Y., Kim J.R., Kee H.J., Kim D.W., Choe N., Park H.J., Son H.J. (2011). Histone methyltransferase SETD3 regulates muscle differentiation. J. Biol. Chem..

[27] Dai S., Holt M.V., Horton J.R., Woodcock C.B., Patel A., Zhang X., Young N.L., Wilkinson A.W., Cheng X. (2020). Characterization of SETD3 methyltransferase-mediated protein methionine methylation. J. Biol. Chem..

[28] Schwartzentruber J., Korshunov A., Liu X.Y., Jones D.T., Pfaff E., Jacob K., Sturm D., Fontebasso A.M., Quang D.A., Tonjes M. (2012). Driver mutations in histone H3.3 and chromatin remodeling genes in paediatric glioblastoma. Nature.

[29] Behjati S., Tarpey P.S., Presneau N., Scheipl S., Pillay N., Van Loo P., Wedge D.C., Cooke S.L., Gundem G., Davies H. (2013). Distinct H3F3A and H3F3B driver mutations define chondroblastoma and giant cell tumor of bone. Nat. Genet..

[30] Lowe B.R., Maxham L.A., Hamey J.J., Wilkins M.R., Partridge J.F. (2019). Histone H3 Mutations: An Updated View of Their Role in Chromatin Deregulation and Cancer. Cancers.

[31] Guo Q., Liao S., Kwiatkowski S., Tomaka W., Yu H., Wu G., Tu X., Min J., Drozak J., Xu C. (2019). Structural insights into SETD3-mediated histidine methylation on β-actin. eLife.

[32] Dai S., Horton J.R., Woodcock C.B., Wilkinson A.W., Zhang X., Gozani O., Cheng X. (2019). Structural basis for the target specificity of actin histidine methyltransferase SETD3. Nat. Commun..

[33] Zheng Y., Zhang X., Li H. (2020). Molecular basis for histidine N3-specific methylation of actin H73 by SETD3. Cell Discovery.

[34] Trievel R.C., Beach B.M., Dirk L.M., Houtz R.L., Hurley J.H. (2002). Structure and catalytic mechanism of a SET domain protein methyltransferase. Cell.

[35] Trievel R.C., Flynn E.M., Houtz R.L., Hurley J.H. (2003). Mechanism of multiple lysine methylation by the SET domain enzyme Rubisco LSMT. Nat. Struct. Biol..

[36] Pettersen E.F., Goddard T.D., Huang C.C., Couch G.S., Greenblatt D.M., Meng E.C., Ferrin T.E. (2004). UCSF Chimera–a visualization system for exploratory research and analysis. J. Comput. Chem..

[37] Dillon S.C., Zhang X., Trievel R.C., Cheng X. (2005). The SET-domain protein superfamily: Protein lysine methyltransferases. Genome Biol..

[38] Raunser S., Magnani R., Huang Z., Houtz R.L., Trievel R.C., Penczek P.A., Walz T. (2009). Rubisco in complex with Rubisco large subunit methyltransferase. Proc. Nat. Acad. Sci. USA.

[39] Chang Y., Levy D., Horton J.R., Peng J., Zhang X., Gozani O., Cheng X. (2011). Structural basis of SETD6-mediated regulation of the NF-kB network via methyl-lysine signaling. Nucleic Acids Res..

[40] Ye S., Ding Y.F., Jia W.H., Liu X.L., Feng J.Y., Zhu Q., Cai S.L., Yang Y.S., Lu Q.Y., Huang X.T. (2019). SET Domain-Containing Protein 4 Epigenetically Controls Breast Cancer Stem Cell Quiescence. Cancer Res..

[41] Jumper J., Evans R., Pritzel A., Green T., Figurnov M., Ronneberger O., Tunyasuvunakool K., Bates R., Žídek A., Potapenko A. (2021). Highly accurate protein structure prediction with AlphaFold. Nature.

[42] Herz H.M., Garruss A., Shilatifard A. (2013). SET for life: Biochemical activities and biological functions of SET domain-containing proteins. Trends Biochem. Sci..

[43] Lukinović V., Casanova A.G., Roth G.S., Chuffart F., Reynoird N. (2020). Lysine Methyltransferases Signaling: Histones are Just the Tip of the Iceberg. Curr. Protein Pept. Sci..

[44] Chen Z., Yan C.T., Dou Y., Viboolsittiseri S.S., Wang J.H. (2013). The role of a newly identified SET domain-containing protein, SETD3, in oncogenesis. Haematologica.

[45] Kudryashov D.S., Reisler E. (2013). ATP and ADP actin states. Biopolymers.

[46] Stemp M.J., Guha S., Hartl F.U., Barral J.M. (2005). Efficient production of native actin upon translation in a bacterial lysate supplemented with the eukaryotic chaperonin TRiC. Biol. Chem..

[47] Frankel A., Brown J.I. (2019). Evaluation of kinetic data: What the numbers tell us about PRMTs. Biochim. Biophys. Acta Proteins Proteom..

[48] Dai S., Horton J.R., Wilkinson A.W., Gozani O., Zhang X., Cheng X. (2020). An engineered variant of SETD3 methyltransferase alters target specificity from histidine to lysine methylation. J. Biol. Chem..

[49] Cohn O., Feldman M., Weil L., Kublanovsky M., Levy D. (2016). Chromatin associated SETD3 negatively regulates VEGF expression. Sci. Rep..

[50] Hintzen J.C.J., Moesgaard L., Kwiatkowski S., Drozak J., Kongsted J., Mecinović J. (2021). β-Actin Peptide-Based Inhibitors of Histidine Methyltransferase SETD3. Chem. Med. Chem..

[51] Bachovchin W.W., Roberts J.D. (1978). Nitrogen-15 nuclear magnetic resonance spectroscopy. The state of histidine in the catalytic triad of .alpha.-lytic protease. Implications for the charge-relay mechanism of peptide-bond cleavage by serine proteases. J. Am. Chem. Soc..

[52] Grimsley G.R., Scholtz J.M., Pace C.N. (2009). A summary of the measured pK values of the ionizable groups in folded proteins. Protein Sci..

[53] Human Protein Atlas. https://www.proteinatlas.org/ENSG00000183576-SETD3/cell.

[54] Sanger J.W., Wang J., Fan Y., White J., Mi-Mi L., Dube D.K., Sanger J.M., Pruyne D. (2017). Assembly and Maintenance of Myofibrils in Striated Muscle. Handb. Exp. Pharmacol..

[55] Su W., Mruk D.D., Cheng C.Y. (2013). Regulation of actin dynamics and protein trafficking during spermatogenesis—Insights into a complex process. Crit. Rev. Biochem. Mol. Biol..

[56] Sever S., Schiffer M. (2018). Actin dynamics at focal adhesions: A common endpoint and putative therapeutic target for proteinuric kidney diseases. Kidney Int..

[57] Cheng X., Hao Y., Shu W., Zhao M., Zhao C., Wu Y., Peng X., Yao P., Xiao D., Qing G. (2017). Cell cycle-dependent degradation of the methyltransferase SETD3 attenuates cell proliferation and liver tumorigenesis. J. Biol. Chem..

[58] Pollard T.D. (2016). Actin and Actin-Binding Proteins. Cold Spring Harb. Perspect. Biol..

[59] Dominguez R., Holmes K.C. (2011). Actin structure and function. Annu. Rev. Biophys..

[60] DiNubile M.J. (1998). Nucleation and elongation of actin filaments in the presence of high speed supernate from neutrophil lysates: Modulating effects of Ca^2+^ and phosphatidylinositol-4,5-bisphosphate. Biochim. Biophys. Acta.

[61] Choua S.Z., Pollarda T.D. (2019). Mechanism of actin polymerization revealed by cryo-EM structures of actin filaments with three different bound nucleotides. Proc. Natl. Acad. Sci. USA.

[62] Solomon L.R., Rubenstein P.A. (1987). Studies on the role of actin’s N tau-methylhistidine using oligodeoxynucleotide-directed site-specific mutagenesis. J. Biol. Chem..

[63] Diep J., Ooi Y.S., Wilkinson A.W., Peters C.E., Foy E., Johnson J.R., Zengel J., Ding S., Weng K.F., Laufman O. (2019). Enterovirus pathogenesis requires the host methyltransferase SETD3. Nat. Microbiol..

[64] Laitinen O.H., Svedin E., Kapell S., Nurminen A., Hytönen V.P., Flodström-Tullberg M. (2016). Enteroviral proteases: Structure, host interactions and pathogenicity. Rev. Med. Virol..

[65] Visser L.J., Langereis M.A., Rabouw H.H., Wahedi M., Muntjewerff E.M., de Groot R.J., van Kuppeveld F.J.M. (2019). Essential role of enterovirus 2A protease in counteracting stress granule formation and the induction of type I interferon. J. Virol..

[66] Pires-Luís A.S., Vieira-Coimbra M., Vieira F.Q., Costa-Pinheiro P., Silva-Santos R., Dias P.C., Antunes L., Lobo F., Oliveira J., Gonçalves C.S. (2015). Expression of histone methyltransferases as novel biomarkers for renal cell tumor diagnosis and prognostication. Epigenetics.

[67] Xu L., Wang P., Feng X., Tang J., Li L., Zheng X., Zhang J., Hu Y., Lan T., Yuan K. (2019). SETD3 is regulated by a couple of microRNAs and plays opposing roles in proliferation and metastasis of hepatocellular carcinoma. Clin. Sci..

[68] Hassan N., Rutsch N., Győrffy B., Espinoza-Sánchez N.A., Götte M. (2020). SETD3 acts as a prognostic marker in breast cancer patients and modulates the viability and invasion of breast cancer cells. Sci. Rep..

[69] Liao G.B., Li X.Z., Zeng S., Liao G.B., Li X.Z., Zeng S., Liu C., Yang S.M., Yang L., Hu C.J. (2018). Regulation of the master regulator FOXM1 in cancer. Cell Commun. Signal..

[70] Xu L., Feng X., Hao X., Wang P., Zhang Y., Zheng X., Li L., Ren S., Zhang M., Xu M. (2019). CircSETD3 (Hsa_circ_0000567) acts as a sponge for microRNA-421 inhibiting hepatocellular carcinoma growth. J. Exp. Clin. Cancer Res..

[71] Tang L., Xiong W., Zhang L., Wang D., Wang Y., Wu Y., Wei F., Mo Y., Hou X., Shi L. (2021). CircSETD3 regulates MAPRE1 through miR-615-5p and miR-1538 sponges to promote migration and invasion in nasopharyngeal carcinoma. Oncogene.

[72] Li Q., Zhang Y., Jiang Q. (2019). SETD3 reduces KLC4 expression to improve the sensitization of cervical cancer cell to radiotherapy. Biochem. Biophys. Res. Commun..

[73] Baek J.H., Lee J., Yun H.S., Lee C.W., Song J.Y., Um H.D., Park J.K., Park I.C., Kim J.S., Kim E.H. (2018). Kinesin light chain-4 depletion induces apoptosis of radioresistant cancer cells by mitochondrial dysfunction via calcium ion influx. Cell Death Dis..

[74] Abaev-Schneiderman E., Admoni-Elisha L., Levy D. (2019). SETD3 is a positive regulator of DNA-damage-induced apoptosis. Cell Death Dis..

[75] Engqvist H., Parris T.Z., Kovács A., Rönnerman E.W., Sundfeldt K., Karlsson P., Helou K. (2020). Validation of Novel Prognostic Biomarkers for Early-Stage Clear-Cell, Endometrioid and Mucinous Ovarian Carcinomas Using Immunohistochemistry. Front. Oncol..

[76] Jiang X., Li T., Sun J., Liu J., Wu H. (2018). SETD3 negatively regulates VEGF expression during hypoxic pulmonary hypertension in rats. Hypertens. Res..

[77] Liao J., Luo S., Yang M., Lu Q. (2020). Overexpression of CXCR5 in CD4^+^ T cells of SLE patients caused by excessive SETD3. Clin. Immunol..

[78] Xu X., Cui Y., Li C., Wang Y., Cheng J., Chen S., Sun J., Ren J., Yao X., Gao J. (2021). SETD3 Downregulation Mediates PTEN Upregulation-Induced Ischemic Neuronal Death Through Suppression of Actin Polymerization and Mitochondrial Function. Mol. Neurobiol..

[79] Garza-Rodríguez M.L., Oyervides-Muñoz M.A., Pérez-Maya A.A., Sánchez-Domínguez C.N., Berlanga-Garza A., Antonio-Macedo M., Valdés-Chapa L.D., Vidal-Torres D., Vidal-Gutiérrez O., Pérez-Ibave D.C. (2021). Analysis of HPV Integrations in Mexican Pre-Tumoral Cervical Lesions Reveal Centromere-Enriched Breakpoints and Abundant Unspecific HPV Regions. Int. J. Mol. Sci..

[80] Gershey E.L., Haslett G.W., Vidali G., Allfrey V.G. (1969). Chemical studies of histone methylation. Evidence for the occurrence of 3-methylhistidine in avian erythrocyte histone fractions. J. Biol. Chem..

[81] Woodcock C.B., Yu D., Zhang X., Cheng X. (2019). Human HemK2/KMT9/N6AMT1 is an active protein methyltransferase, but does not act on DNA in vitro, in the presence of Trm112. Cell Discov..

[82] Gao J., Wang B., Yu H., Wu G., Wan C., Liu W., Liao S., Cheng L., Zhu Z. (2020). Structural insight into HEMK2-TRMT112-mediated glutamine methylation. Biochem. J..

